# Metal–Organic Frameworks for Electrocatalytic Sensing of Hydrogen Peroxide

**DOI:** 10.3390/molecules27144571

**Published:** 2022-07-18

**Authors:** Shuhan Wang, Tong Zhang, Xukun Zhu, Shu Zu, Zexin Xie, Xiaoxiang Lu, Mingdao Zhang, Li Song, Yachao Jin

**Affiliations:** Jiangsu Key Laboratory of Atmospheric Environment Monitoring and Pollution Control, Jiangsu Collaborative Innovation Center of Atmospheric Environment and Equipment Technology, Institute of Energy Supply Technology for High-End Equipment, School of Environmental Science and Engineering, Nanjing University of Information Science & Technology, Nanjing 210044, China; wsh2638237340@outlook.com (S.W.); zhangtong9696@163.com (T.Z.); xukunzhu7@gmail.com (X.Z.); zushu_zs@163.com (S.Z.); xiezexin2001@163.com (Z.X.); l1214490676@163.com (X.L.); songli@nuist.edu.cn (L.S.); jinyachao@nuist.edu.cn (Y.J.)

**Keywords:** H_2_O_2_, MOFs, electrochemical detection, sensors

## Abstract

The electrochemical detection of hydrogen peroxide (H_2_O_2_) has become more and more important in industrial production, daily life, biological process, green energy chemistry, and other fields (especially for the detection of low concentration of H_2_O_2)._ Metal organic frameworks (MOFs) are promising candidates to replace the established H_2_O_2_ sensors based on precious metals or enzymes. This review summarizes recent advances in MOF-based H_2_O_2_ electrochemical sensors, including conductive MOFs, MOFs with chemical modifications, MOFs-composites, and MOF derivatives. Finally, the challenges and prospects for the optimization and design of H_2_O_2_ electrochemical sensors with ultra-low detection limit and long-life are presented.

## 1. Introduction

Hydrogen peroxide (H_2_O_2_) is an indispensable component in living organisms as a biological intermediary, and has been widely applied as an essential reagent in daily life, industrial fields, medical treatment, and other sections. However, a high concentration of H_2_O_2_ is corrosive and can cause serious injury when contacts with the skin, and accidental ingestion of H_2_O_2_ can result in gas embolism in several organs, especially in heart, lung and esophagus [1,2,3,4]. The strong oxidizing property of H_2_O_2_ can even be applied for the preparation of explosives, which may threaten people’s lives [5,6]. More importantly, the harm caused by low-concentration H_2_O_2_ should not be underestimated. Actually, trace amounts of H_2_O_2_ produced in the cells can cause the induced oxidation of melanocytes, resulting in the death of melanocytes, which is also the main cause of vitiligo [7]. Abnormal production of H_2_O_2_ in mitochondria can cause reversible mitochondrial swelling, rupture and cellular structural changes, which can induce diseases such as diabetes, Parkinson’s diseases and cancers [8]. Therefore, it’s of great significance to detect low concentration of H_2_O_2_.

For now, a great number of H_2_O_2_ detection approaches, such as electrochemical detection [9], fluorescence [10], titration [11] and chromatography [12] have been employed in detecting H_2_O_2_ concentration. However, traditional methods are no longer applicable in many fields with the higher requirements for H_2_O_2_ detection. For instance, precision data and low detection limit can be obtained through fluorescence detection, while high detection cost and requirement of sample preparation remain a problem [13]. Compared with fluorescence detection method, the colorimetric detection method itself has the advantages of low cost and fast detection speed. However, the raw materials can be easily denatured which makes it difficult to prepare [14]. In contrast, electrochemical detection method has been widely used owing to its advantages of simplicity, rapidity, sensitivity and economy [15,16]. Despite that, electrochemical H_2_O_2_ detection usually requires a higher potential for direct reduction or oxidation on the surface of the bare electrode, resulting in a less current response. Therefore, it is essential to improve the bare electrode or design new electrode materials to increase the sensitivity of the H_2_O_2_ sensor and speed up the response time for the detection of ultra-low concentration of H_2_O_2_ [17].

## 2. Research Status and Challenges

Hitherto, various strategies have been reported to modify the electrodes. Among them, the most common one is to prepare the metal oxide modified electrodes. It has been observed that the electrocatalytic materials containing Fe_2_O_3_ show excellent catalytic performance, where the detection limit reaches 5 nmol, and they can be applied to detect H_2_O_2_ both inside and outside the cells [18,19,20]. Apart from Fe_2_O_3_, Cu_2_O features high specific surface area, good electrochemical activity and the potential to promote electron transfer reactions at a lower overpotential, making it a favorable material for the development of H_2_O_2_ sensors. Nevertheless, the catalytic activity is affected by the particle size and the shape of the metal oxides. On the whole, the large particle size and irregular morphology reduce the effective contact between the catalytic materials and the H_2_O_2_ molecules, resulting in incomplete catalytic activity and reduced catalytic effect [21,22]. Additionally, enzymes are often used to modify the electrodes due to their perfect selectivity for H_2_O_2_. Horseradish peroxidase (HRP), as one of the most common H_2_O_2_ enzymes, although has been extensively studied and used for manufacturing H_2_O_2_ sensors [23], its application has been restricted by the complex immobilization process and the instability during the detection [24]. Currently, precious metal nanoparticles (Pt, Ag, Ru et al.) have been widely implemented in H_2_O_2_ detection technology due to their unique electronic structure, prominent physical and chemical properties [25]. For instance, AuNCs (nanoclusters) is an emerging nanomaterial that exhibits excellent performance and can improve the sensitivity of H_2_O_2_ detection [26]. B. Patella and co-workers designed the rGO/Au-NPs (nanoparticles)-based electrode through a three-electro deposition method to monitor H_2_O_2_ released by the human macrophages [27]. However, considering the scarcity of precious metals, it is necessary to find and develop new materials with low cost and high catalytic activity to replace precious metals [28,29].

**Figure 1:**
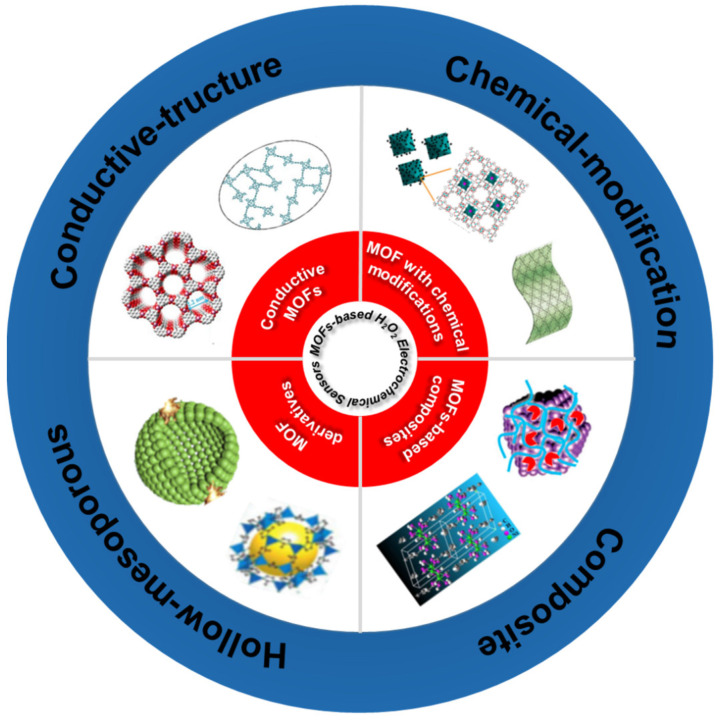
Conductive MOFs based H_2_O_2_ sensors; chemically modified MOFs-based H_2_O_2_ sensors; MOFs composites based H_2_O_2_ sensor; MOF derivatives based H_2_O_2_ sensor.

In recent years, numerous new materials have also been used for electrochemical detection of H_2_O_2_, such as CNTs (carbon nanotube), graphene and metal organic frameworks (MOFs) [30,31,32]. As we know, the electrical conductivity and catalytic performance can be improved by the carbon coating method; when the graphene oxide nanoflake is covered by nano-scale graphene, the subsequently synthesized graphene oxide/graphene composite material presents a better performance on H_2_O_2_ detection with high catalytic activity and electrochemical stability [33]. Yu et al. [34] developed a novel/graphene (NiO/GR) nanocomposite that exhibited high sensitivity to H_2_O_2_, the GR coating improved the electrochemical stability and its anti-interference ability. MOFs have drawn increasing interest and been applied to gas storage/separation [35,36,37,38,39,40,41,42,43,44], drug delivery [35,36,37,38,39,40,41,42,43,44], energy storage/conversion [35,36,37,38,39,40,41,42,43,44] and multiphase catalysis [35,36,37,38,39,40,41,42,43,44] due to their tunable pore sizes, diverse structures, and abundant functional designs [35,36,37,38,39,40,41,42,43,44]. Moreover, the ordered arrangement of metal sites and organic ligands provide abundance of accessible catalytic sites and confer intrinsic enzyme-mimetic properties to MOFs compared to other types of nanozymes [45]. For example, Zhang [46] developed the new class of 2D conductive MOFs films [Co_3_(HHTP)_2_]*_n_* by LB (langmuir-blodgeet) technology. The porous structures and exposed Co active sites from the [Co_3_(HHTP)_2_]*_n_* had superior catalytic activity for H_2_O_2_. In addition, some MOFs with mesh structures can be used as carriers for other materials to form composite materials with new functions [46]. There have been several review reports on the use of MOFs for H_2_O_2_ sensors [45,47,48,49,50,51,52], for example, Li et al. [45,47,48,49,50,51,52] classified MOFs into four categories and reviewed the progress of MOFs in chemical sensors, including the detection of H_2_O_2_. Goncalves et al. [45,47,48,49,50,51,52] reviewed the development of MOFs derivatives for sensors. However, most of these reports are not comprehensive enough to introduce all of the types of MOFs-based H_2_O_2_ sensors. What’ more, instead of focusing on H_2_O_2_ detection, variety of molecules are also included, which makes most of these reviews not deeply explore the mechanism of MOFs for H_2_O_2_ sensing, as well as the design and development of MOFs H_2_O_2_ electrochemical sensors. In this paper, based on the reviews of the same topic, as well as the latest research progress, we found that MOFs can exert more structural advantages in electrochemical detection of H_2_O_2_ (strong adsorption, faster electron transport, and high conductivity) and become more competitive as a sensor. Here, we deeply explore the detection mechanism of electrochemical H_2_O_2_ for different types of MOFs, some bright ideas for the design and improvement of MOFs-based electrochemical H_2_O_2_ sensors are proposed based on the recent research progress. Firstly, the sensing mechanism and working principle of MOFs-based H_2_O_2_ electrochemical sensors are distinctly introduced. Secondly, as shown in Figure 1, we categorize the recent reported MOFs-based H_2_O_2_ electrochemical sensors as: (1) conductive MOFs based H_2_O_2_ sensors; (2) chemically modified MOFs based H_2_O_2_ sensors; (3) MOFs composites based H_2_O_2_ sensor; (4) MOF derivatives based H_2_O_2_ sensors. Finally, problems and trends for future MOFs-based H_2_O_2_ electrochemical sensors are discussed. We hope that our perspectives will be useful for future development of advanced MOF-based H_2_O_2_ sensors. In addition, the performance of recently reported MOFs-based H_2_O_2_ electrochemical sensors are shown in Table 1. We also summarized the following characteristics as the keys to develop MOFs-based H_2_O_2_ electrochemical sensors with excellent performance:

(i) Conductivity

High electrical conductivity is vital to electron transfer in the process of H_2_O_2_ splitting. In general, the electrical conductivity of MOFs depends on the carrier mobility and internal charge density. The π-conjugated structure forms a continuous conductive network to facilitate the conduction of electrons. In addition, high-energy electrons or holes in metal ions can induce high concentrations of loosely bound carriers, thereby increasing the charge density. The direct connection between the metal nodes and the organic ligands can effectively reduce the energy mismatches and further promote the charge delocalization and electron transfer.

(ii) Activity

The catalyst activity is an important factor in the decomposition rate of H_2_O_2_. It is mainly related to the intrinsic catalytic ability and the number of active sites of the catalyst. Generally, the porous structure can not only increase the specific surface area, but also expose more active sites to improve the catalytic activity, thus further improve the adsorption capacity, reduce the activation energy of the intermediate product transformation.

(iii) Selectivity

An ideal electrochemical sensor for H_2_O_2_ should have high selectivity. For example, H_2_O_2_ samples may be mixed with other substances (glucose, ascorbic acid, ethanol, etc.), and they will also undergo redox reactions. Therefore, the sensors should only respond to H_2_O_2_ at a specific voltage.

(iv) Stability

The commercial H_2_O_2_ electrochemical sensors must possess excellent chemical and structural stability in hostile acidic and alkaline environments. In specific cases, it may suffer from changes in catalyst structure and reduced catalytic activity due to the particle agglomeration during the long-time detection process. Therefore, in order to ensure the accuracy of the test, especially in medical clinical applications, the requirement of high stability need be met in the design of commercialized sensors.

(v) Low-cost

Precious metal electrocatalysts (e.g., Pt, Au, Ag, Pd) have high catalytic activity, but their scale-up application is limited by high cost, limited reserves, poor stability, low sensitivity and selectivity. Alternatively, MOFs-based catalysts are commonly ligated with transition metals, which have inherently good catalytic activity and can be feasibly prepared by simple methods (hydrothermal method, ultrasonic method et al.). Thus, in order to meet the goal of commercial development, the raw materials and preparation methods of the H_2_O_2_ electrochemical sensors should follow the principles of low cost and low time consumption.

(vi) Environmental-friendly

The concept of low-carbon green development is of great significance to the sustainable development of human society. Specifically, the raw materials for the H_2_O_2_ electrochemical sensors should be non-toxic and non-polluting. The preparation process should not produce hazardous gases and solutions. What’s more, the catalysts must be non-hazardous to the cells, in order to meet the medical detection requirements.

## 3. Sensing Mechanisms and Working Principles of MOFs-Based H_2_O_2_ Electrochemical Sensor

### 3.1. Electrochemical Sensor Detection Principle

**Figure 2:**
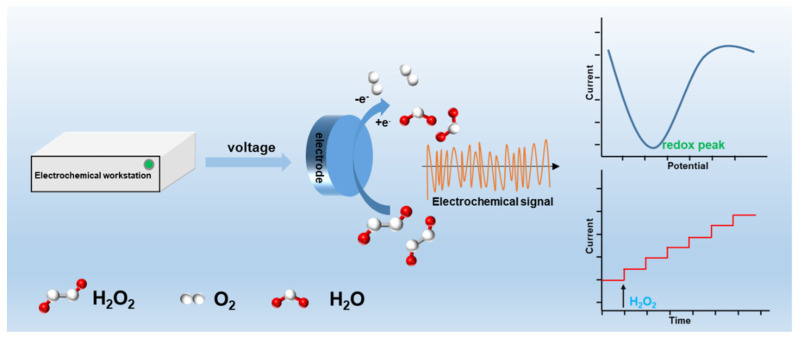
Schematic diagram of H_2_O_2_ detection by electrochemical sensor.

Generally, the electrochemical detection of H_2_O_2_ is achieved by applying a corresponding voltage to the electrochemical sensor, followed by the oxidation or reduction of H_2_O_2_ on the electrode surface. The electric charges of the redox process are captured by the electrode and converted into an electrochemical signal. Qualitative and quantitative analysis of H_2_O_2_ can be obtained through the change in the peak voltage and current. The schematic diagram of electrochemical detection of H_2_O_2_ is shown in Figure 2. Commonly, H_2_O_2_ detection is performed under neutral or alkaline conditions, with certain differences in the reaction mechanism. Specifically, the difference lies in whether the catalyst produces a reducing or oxidizing effect on H_2_O_2_ during the detection: if the catalyst appears in an oxidized state, the H_2_O_2_ is oxidized; and if the catalyst is in a reduced state, the H_2_O_2_ is reduced. We will discuss the specific steps of H_2_O_2_ catalytic decomposition in the following sections.

### 3.2. Detecting under Alkaline Condition

Under alkaline conditions, the catalytic decomposition of H_2_O_2_ mainly depends on the redox changes of metal sites. In the first case, the catalyst combines with OH^−^ to form intermediates with higher valence state of the metal sites and electrons, then the intermediates react with H_2_O_2_ to return to the original valence state, and H_2_O_2_ is decomposed. The mechanism can be illustrated by the following equation [80]:

(1) $$\left[\left(adp\right)\left(BIB\right)Cu^{II}-OH_{2}\right]+OH^{-}⇄\left[\left(adp\right)\left(BIB\right)Cu^{III}-OH\right]+H_{2}O+e^{-}$$

(2) $$\left[\left(adp\right)\left(BIB\right)Cu^{III}-OH\right]+1/2H_{2}O_{2}\to \;\left[\left(adp\right)\left(BIB\right)Cu^{II}-OH_{2}\right]+1/2O_{2}$$

The other situation is that H_2_O_2_ is adsorbed by catalysts, then the catalysts are reduced as the cathode. After that, the pre-adsorbed H_2_O_2_ is decomposed by electron transfer with the catalyst. The mechanism can be illustrated by the following equation [80]:

(3) $$MIL-53-Cr^{III}+e^{-}\to MIL-53-Cr^{II}$$

(4) $$MIL-53-Cr^{II}+H_{2}O_{2}\to MIL-53-Cr^{III}+H_{2}O+O_{2}$$

### 3.3. Detecting under Neutral Condition

It has been reported that the catalytic decomposition of H_2_O_2_ occurs in the presence of ferrous (Fe^2+^) and ferric (Fe^3+^) ions. The generation of reactive hydroxyl radicals (HO·) is based on the classical Haber Weiss mechanism/Fenton-type reaction [81,82,83,84]. The results show that ferrous ions have better catalytic decomposition activity for H_2_O_2_ in the pH range of 3–4, while ferric ions have better catalytic activity at a neutral pH. The reactivity study of ferric ions show that the coordinated ferrous ions catalyze the decomposition of H_2_O_2_ more effectively than the free ferric ions at the neutral pH [82]. In this case, the ferric ions are first reduced into ferrous, and then the ferrous ions are oxidized by H_2_O_2_ to turn to ferric ions. The electrochemical mechanism of general Fe-MOFs based materials in neutral media are as follows [85]:

(5) $$Fe^{3+}+e^{-}\to Fe^{2+}$$

(6) $$Fe^{2+}+H_{2}O_{2}\to Fe^{3+}+HO^{-}+HO^{-}$$

(7) $$O^{\cdot }+e^{-}\to HO^{-}$$

(8) $$HO^{-}+H^{+}⇌H_{2}O$$

## 4. Design and Synthesis of MOFs Based H_2_O_2_ Sensor

MOFs are a kind of porous polymer materials connected by metal ions through organic bridge ligands, which combine the advantages of inorganic, organic porous materials and porous hybrids [86,87,88,89]. The structural diversity makes some MOFs possess the multi-channel structures which can enhance the electrolyte transport capacity and further increase the electrical conductivity. Furthermore, the reactants can be easily trapped and adsorbed by various active sites and high specific surface areas, thus the conversion of the reaction is facilitated [90]. Therefore, MOFs have great prospect for the preparation of H_2_O_2_ sensors. In the following sections, some reliable ideas to flexibly apply MOFs materials to the design of H_2_O_2_ sensors are expounded.

### 4.1. Conductive MOFs Based H_2_O_2_ Sensor

Ionic conduction and electron conduction occur along with the H_2_O_2_ decomposition in the process of detection. However, the conversion efficiency between electrical and chemical energy will be reduced if the conductive orbit cannot be effectively constructed, and leading to decreasing adsorption and catalytic capacity [91]. In general, the formation of conductive network requires coordination interaction between metal ions and organic ligands. Ligands with π-conjugated structures can form a continuous conductive network to facilitate electron conduction. High concentration of loose carriers formed by high-energy electrons or holes in metal ions can increase charge density [91]. The direct connection between metal nodes and organic ligands (such as N, O, S-based ligands) can effectively reduce the energy mismatch, further promoting the charge delocalization and electron transport [92,93,94]. Based on the charge transfer mode, the conductive mechanism of conductive MOFs can be divided into three types as follow: charge transfer “through space”, through-bond conduction and through-guest conduction [95].

**Figure 3:**
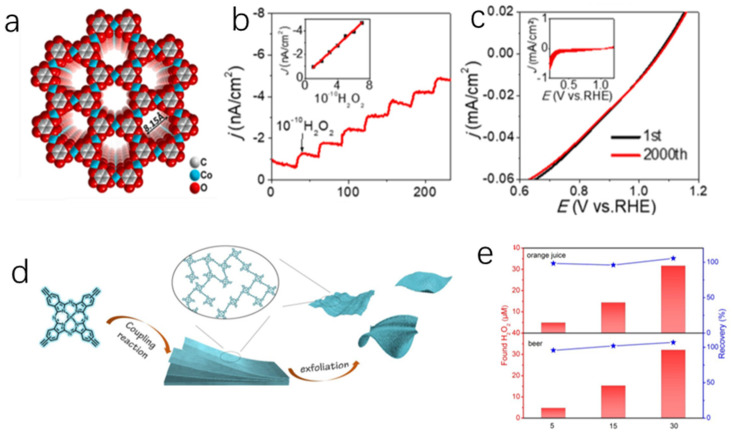
(**a**) The space-filling model of [Co_3_(HOB)_2_]*_n_*; (**b**) H_2_O_2_ injection responses of the current density of 3-layer [Co_3_(HOB)_2_]*_n_* films (The inset shows the linear relationship between the current density and the injected H_2_O_2_ amount.); (**c**) Tolerance of regular species. LSV curves before/after 1000 CV cycles. (The inset shows the 1000-cycle CV curves.) [36]; Copyright 2021, Elsevier. (**d**) Schematic diagram for the synthesis of the FePc-CP NSs; (**e**) Determination of H_2_O_2_ in commercial orange juice and beer [55]. Copyright 2019, ACS.

Aromatic hydrocarbons are a class of compounds with conjugated structures, such as 2,3,6,7,10,11-hexaiminotriphenylene (HITP), 2,3,6,7,10,11-hexahydroxytriphenylene (HHTP) and benzenehexol (HOB). These π-conjugated aromatic systems are capable of cooperating with metal nodes to dominate the conduction process. The π-π interaction between the systems and metals facilitates both “through-bond” and “through-space” electronic conduction [96,97]. Our group [36] designed the 2D [Co_3_(HOB)_2_]*_n_* conductive MOF nanosheets with abundant nanoscale channels. HOB and Co^2+^ ions were coordinated at the water-air interface to form a single-layer nanosheets with high structural order through LB method (Figure 3a). [Co_3_(HOB)_2_]_n_ nanosheets were arranged in a long-range order, and then the π-π stacks in the internal pores were formed by original growth on the FTO (fluorine-doped tin oxide) glass. Meanwhile, the coordination of Co-O reduced the energy mismatch, facilitated the charge delocalization and electron transport. The counterion pair generated from electrostatic interactions leads to the feasible electronic adjustment and migration [98,99]. More importantly, the porous structure for proton transport and the exposed metal sites equip the 3-layer nanosheets with excellent reducibility to H_2_O_2_ with a LOD (limit of detection) of 3.08 nΜ (Figure 3b), and it possesses the lowest detection limitation among the current non-precious metal conductive MOFs based H_2_O_2_ sensors. It could also be concluded that the common drugs and ion concentration had no impact on H_2_O_2_ detection. Noticeably, the activity of [Co_3_(HOB)_2_]*_n_* exhibited almost no attenuation after 1000 CV cycles (Figure 3c). Park [80] et al. reported a Co-based 2D conductive MOF, Co-HAB. The high conductivity (1.57 S cm^−1^), porosity and high density of redox active sites of Co-HAB provided a possibility for ion storage and energy conversion, which may improve the catalytic activity when used for H_2_O_2_ reduction. At the same time, excellent chemical and thermal stability increase the utility of sensors. This offers a bright future for the preparation of H_2_O_2_ sensors with low detection limit.

Coordination bonds composed of metals and organic ligands with matched energy levels and good orbital overlap can generate long-distance charges transfer path, which is beneficial for improving the charge transfer [100]. Liu [55] reported an advanced FePc (iron phthalocyanine) -based diyne-linked (−C≡C−C≡C−) conjugated polymers 2D NSs (nanosheets) (FePc-CP NSs) (Figure 3d). It has been demonstrated that the high reduction activity of the catalyst for H_2_O_2_ detection (LOD was 0.017 μM) is mainly attributed to the following points: (1) FePc is beneficial to promote the cleavage of O-O bonds between O_2_ and peroxide and thereby accelerates the decomposition of H_2_O_2_; (2) diyne-linked (−C≡C−C≡C−) conjugated structure enhances the conductivity; (3) highly exposed heme-like active centers in layered pore junctions increase catalytic activity. FePc-CP NSs accurately detected H_2_O_2_ in beer and orange juice with a recovery of 95.8~107% (Figure 3e), this showed that the FePc-CP NSs had the potential to detect the H_2_O_2_ in food. Since then, we can conclude that long-range ordered nanochannels formed by the accumulation of 2D π-conjugates can realize the electrolyte transport. This approach to creating the charge transport by using covalent bonds of extended coordination polymers in porous frameworks provides a unique platform for the development of high sensitive H_2_O_2_ sensors.

Apart from the above mentioned, improving the concentration of proton carriers is a key factor to construct highly conductive and stable MOFs. The most effective strategy is to introduce the Lewis acid particles [101,102], molecules or counter ions [103,104] and the incorporation of proton-related substituents (–NH_2_, –SO_3_H, –COOH, –OH, –SH, etc.) in the ligands [105,106]. The increasing number of these guest molecules and proton carriers will form a hydrogen bonding network and enhance the conductivity [107,108]. Zhang [109] and coworkers bridged Cu^2+^ with BIB and adp^2−^ to form a 2D propagation network with channel structure. The 2D layer channel structure was further connected by the hydrogen-bond interactions, and then a 3D supramolecular architecture ([Cu(adp)(BIB)(H_2_O)]_n_) was formed. [Cu(adp)(BIB)(H_2_O)]_n_ performed a high reduction for H_2_O_2_ with a low LOD (0.068 μM) and an excellent linear range (0.1 μM to 2.75 μM), which could be attributed to the electronic transport effect. Throughout the redox reaction, the channels and hydrogen-bond acted as transport corridors. Protons and electrons were transferred in an ordered manner. Consequently, the adsorption energy of the active sites for electrolytes and intermediates was decreased. Judging from the electron transfer, it was a two-step reaction. Firstly, [Cu(adp)(BIB)(H_2_O)]*_n_* reacts with OH^−^ and then Cu^2+^ is oxidized to Cu^3+^; secondly, H_2_O_2_ molecules react with Cu^3+^ and then they are reduced to form O_2_ due to the strong oxidizing from Cu^3+^.

Conductive electrical system consisted of metal ions and ligands with π-conjugate structure solve the serious poor conductivity issue for H_2_O_2_ electrocatalysts; furthermore, the porous structure is conducive to promoting the mass transfer, and improving the catalytic rate of H_2_O_2_ decomposition. Still, the high price of organic ligands remains a large obstruction on the pathway to large-scale commercialization.

### 4.2. Chemically Modified MOFs Based H_2_O_2_ Sensor

Chemical modification is a feasible and effective way to introduce desired functions into MOFs materials. In general, MOFs can be functionally modified by their metal sites and/or organic linkers. For example, through the hybridization with conducting polymers, the electrical conductivity of the material can be improved [45,110].

**Figure 4:**
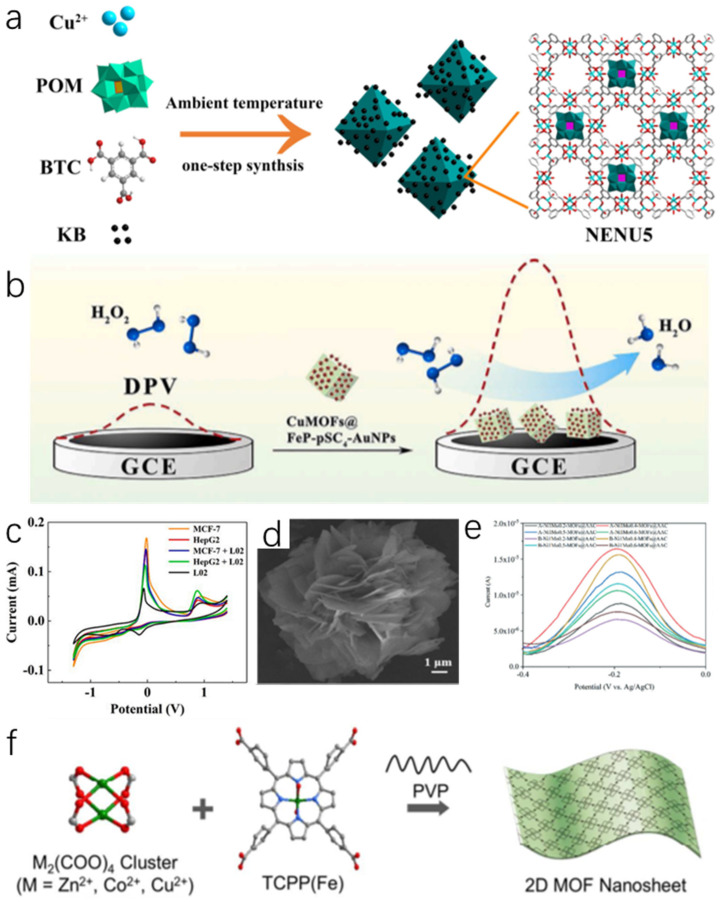
(**a**) Schematic illustration of the synthesis procedure for NENU5-KB composites [58]; Copyright 2018, Wiley. (**b**) Schematic illustration of the electrocatalytic reaction of H_2_O_2_ catalyzed by CuMOFs@FeP-pSC_4_-AuNPs; (**c**) CV responses of H_2_O_2_ with different cells [57]; Copyright 2021, Elsevier. (**d**) SEM image of Ni–MOFs [9]; Copyright 2019, Elsevier. (**e**) DPV curves of A(B)-NixMoy-MOFs@AAC sensors [63]; Copyright 2020, Royal Society of Chemistry. (**f**) Scheme showing the surfactant-assisted bottom-up synthesis of 2D MOF nanosheets [127]. Copyright 2017, ACS.

Combining MOFs with conductive species can improve electronic conductivity, catalytic activity, and expand applications. The most common method is to combine the active component with carbon-based materials such as carbon nanotubes and ketone black carbon [111]. POMs (polyoxometalates), with internal unconventional molecular structure, stable physical/chemical properties and redox state, have shown superb activity in fabricating electrocatalysis. When they are used to prepare MOFs, the latest material POM-based MOFs (POMOFs) performed high specific surface and exposed more active sites [29,112,113]. However, the low conductivity reduces the catalytic activity of POMOFs as catalysts, resulting in low sensitivity and high detection limits when applied for H_2_O_2_ detection. Wang et al. [58] developed POM-based MOF (NENU5 (polyoxometalate-based metal-organic framework)) grown in situ on KB by adopting a facile and feasible one-step solution method (Figure 4a). The low detection limit (1.03 μM) of NENU5-KB-3 for H_2_O_2_ is mainly attributed to the high conductivity of KB and the high catalytic activity of NENU5. It also had a wide linear range from 10 μM to 50 mM and a high sensitivity of 33.77 μA mM^−1^. More notably, the residual current decreased by ~9% after continuous testing in 30 μM H_2_O_2_ for 4 h. It indicated NENU5-KB-3 had good structural stability and stable catalytic activity. Furthermore, drug resistance results have revealed that the successive addition of 0.2 mM AA, 0.2 mM APAP, 0.2 mM DA and 0.2 mM glucose exerted no influence on H_2_O_2_ detection, representing excellent stability and high selectivity.

Conductive substrates such as carbon cloth, FTO, ITO [112,113,114], etc. are also often used as supports for MOFs. They can improve the conductivity while exposing more active components, greatly improving the catalytic activity. Xia [56] and coworkers synthetized Co-MOF nanosheets array supported on Ti mesh (Co-MOF/TM). Co-MOF was synthesized by coordination of Co^2+^ with terephthalic acid. With notable properties of large surface area, good stability, high porosity, and rich unsaturated Co^2+^ sites, Co-MOF/TM exhibited outstanding catalytic performance with a low detection limit (0.25 μM). The catalytic activity can be attributed to the appearance of electronic defects on the surface of the nanosheets arising from the interaction between the Ti lattices and the Co-MOF. The sensor also showed good utility for monitoring the release of H_2_O_2_ from A495 cells. Compared with most MOFs, Co-MOF had a lower cost in both raw materials and the preparation process.

The prospective availability of free space in MOFs permits another method of inducing conductivity, namely the introduction of guests (electron donors or acceptors). In some cases, they are complementary to the charge transfer of MOFs junctions or nodes. This approach not only enables the manufacture of conductive MOFs, but also allows their conductive properties to be modulated by specific stimuli without destroying their structure [95]. Hu et al. [57] assembled the petal-like nanonetwork structures by embedding the combination of pSC_4_-AuNPs and FeP on the surface of CuMOFs (Figure 4b). On the basis of maintaining the original catalytic activity, selective functionalization and well-defined configurations of CuMOFs, FeP (guest) and pSC_4_-AuNPs (body) were interlinked to form a nanonetwork structure for synergistic electron transfer to overcome the drawback of poor conductivity. The dual metallic active sites from the CuMOFs@FeP-pSC4-AuNPs increased the peroxidase activity, electrocatalytic activity and provided good thermal and storage stability. From which, it realized specific recognition of cancer cells by the signal output from the electronic transfer generated of H_2_O_2_ decomposition, providing potential applications for clinical cancer detection (Figure 4c).

Transition metal elements are crucial components to build active adsorption sites in MOFs structures. Among them, Ni, Co and Fe show high activity in the kinetics of H_2_O_2_ decomposition [53,115]. Ni ion has become an important non-precious metal ion for the detection of H_2_O_2_, and the substantial coordination number makes it easy to coordinate with organic ligands [116]. It is worth noting that the dispersion of unsaturated metal sites in MOFs plays an important role in the kinetic activity of H_2_O_2_ decomposition [117]. Therefore, the introduction of another metal can produce more active sites to improve the electrochemical properties. In general, the synergistic interaction of two or more substances effectively improves electrocatalytic performance. This optimizes the electronic structure, reduces kinetic barriers and improves the charge transfer between the host metal atoms and the dopants during catalysis [118,119,120]. Li et al. [63] reported a new bimetals MOF (A(B)-Ni_1_Mo_0.5_-MOFs@AAC) by the liquid-phase and hydrothermal methods. Mo exhibited a strong hydrogen binding capacity and had a higher activity in the decomposition of H_2_O_2_ [121]. What’s more, the introduction of Ni-Mo bimetals enhanced the stability of the frameworks, and contributed to the catalytic performance (Figure 4e). Therefore, the A(B)-Ni_1_Mo_0.5_-MOFs@AAC exhibited an outstanding detection performance for H_2_O_2_ with a high sensitivity (0.277μA μM^−1^) and a noteworthy low detection limit of 0.185 μM. The establishment of bimetallic MOFs offers a new and simple idea for designing H_2_O_2_ sensors with high structural stability and excellent catalytic activity.

Today, widely reported MOFs such as MIL (materials of institut lavoisier)-53(Fe), Fe-NH_2_-MIL-88, MIL-68(Fe), and MIL-100(Fe), etc. all exhibit salient H_2_O_2_ decomposition properties. The narrow pore channels from the 3D block crystal structure restrict the diffusion rate of the substrate, and they further reduce the accessible active site in the 3D native crystal [46,122]. The special structure of 2D nanomaterials, such as large specific surface area, thin thickness and high surface volume, can expose more active sites on the surfaces, thus reducing the mass transfer resistance and diffusion potential barrier [123,124,125,126]. Wang et al. [127] synthesized a 2D bimetallic MOF nanosheets (2D Co-TCPP (tris (1-chloro-2-propyl) phosphate) (Fe)) with a thickness of less than 10 nm for the first time through a surface activity-assisted approach (Figure 4f). The highly exposed active sites enabled 2D Co-TCPP(Fe) to exhibit excellent H_2_O_2_ catalytic decomposition activity with a detection limit of 0.15 × 10^−6^ M. In addition, the sensor was successfully used for the real-time monitoring of H_2_O_2_ secreted by living cells. The synthesis of 2D Co-TCPP(Fe) offers a versatile method for developing 2D bimetallic MOF nanosheets in high yields, which can be applied to a variety of areas.

### 4.3. MOF Composites Based H_2_O_2_ Sensor

**Figure 5:**
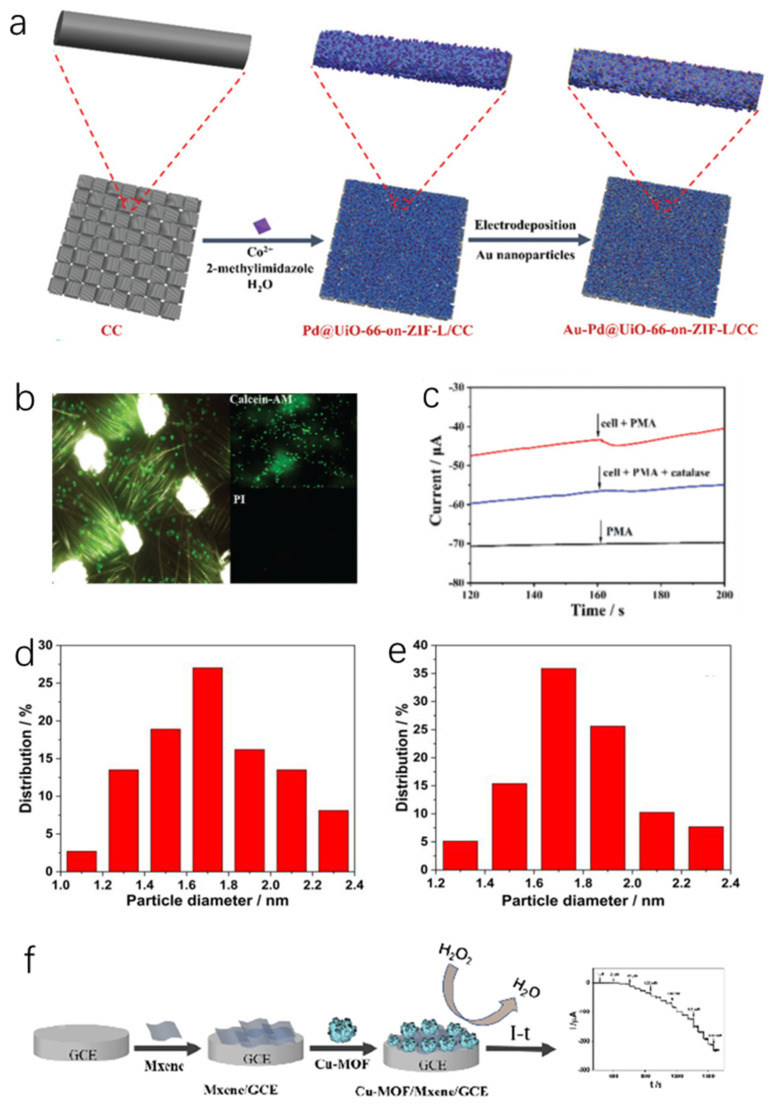
(**a**) Schematic diagram of the synthesis of Au–Pd@UiO-66-on-ZIF-L on CC; (**b**) Fluorescence spectra of A549 cells grown on Au–Pd@UiO-66-on-ZIF-L/CC stained by Calcein-AM (green) and PI (red); (**c**) Amperometric responses of the Au–Pd@UiO-66-on-ZIF-L/CC electrode to H_2_O_2_ secreted from living A549 cells under drug stimulation at 0.6 V [67]; Copyright 2021, Royal Society of Chemistry. (**d**) Particle size distributions of AgNPs in AgNPs@Y-1,4-NDC-MOF/ERGO and (**e**) CuNPs in CuNPs@Y-1,4-NDC-MOF/ERGO [62]; Copyright 2018, Elsevier. (**f**) The fabrication of the electrochemical sensor for the detection of H_2_O_2_ [64]. Copyright 2021, Wiley.

Metal nanoparticles are a kind of economic, stable, simple to prepare nanomaterials, which have similar enzyme action to specific molecules [128,129]. In particular, “d” electron orbitals of precious metal nanomaterials are not filled, the surface may become easier to adsorb reactants. The moderate strength facilitates the formation of intermediate “active compounds” with high catalytic activity and excellent properties such as high temperature resistance, oxidation resistance and corrosion resistance [130,131]. Moreover, the active sites on their surface give them properties similar to those of biological enzymes [128,129]. Since MOFs have a permanent porous structure, the advantages of nanoparticles and MOFs can be utilized by introducing specific nanoparticles into MOFs to form a more stable material [132]. Shazia et al. [67] obtained a bimetallic MOF (Au-Pd@UiO-66-on-ZIF (zeolite imidazolium ester skeleton structure material) -L/CC) by introducing Au nanoparticles into Pd@UiO-66-on-ZIF-L/CC (Figure 5a). Electrochemical test results showed that the introduction of Au nanoparticles increased the adsorption sites of H_2_O_2_, and the synergistic effect between Au nanoparticles and Pd improved the catalytic performance (LOD was 21.2 nM), anti-interference, reproducibility, repeatability and stability of H_2_O_2_ sensor. Meanwhile, real-time in situ detection of H_2_O_2_ was achieved by culture of human adenocarcinoma alveolar basal epithelial cells (A549 cells) on Au-Pd@UiO-66-on-ZIF-L/CC, suggesting that the sensor has potential applications in cancer pathology (Figure 5b,c). Li et al. [62] prepared MNPs (magnetic nanoparticles) @Y-1, 4-NDC-MOF/ERGO (M = Ag, Cu) ternary composites by cation exchange strategy and electrochemical reduction. The embedding of metal nanomaterials improved the catalytic activity of the material, while the fast electron transfer effect of ERGO increased the electrical conductivity. What’s more, the size tunability and selectivity (Figure 5d,e) of MOFs provided the material with high selectivity to H_2_O_2_. The material had potential applications in detecting the release of H_2_O_2_ from cells. The interaction between the active component and the supporter plays a pivotal role in the catalytic reaction. On the one hand, uniform dispersion of metal nanoparticles can effectively increase the specific surface area while the unity of metal nanoparticles will minimize the specific surface area and surface energy, resulting in a severe loss of structural properties [131]. In order to increase the catalytic activity by loading more metallic nanoparticles, suitable carriers (crystals with high chemical stability, large specific surface area and high porosity) are preferably selected to disperse and immobilize metal particles [133,134]. Based on the large specific surface area, sufficient pore capacity and excellent crystallinity of MOF-67, Wang et al. [32] uniformly dispersed Au@Pt bimetallic nanoflowers on its surface. Compared with the single metal materials, Au@Pt with core-shell structure of bimetallic nanoflowers showed abundant active sites, good electrical conductivity, and better H_2_O_2_ catalytic activity. The large specific surface area of the MOFs material provides more loading sites for more nanoparticles, thus further enhancing the catalytic effect. They have been proven to be a powerful electrochemical sensing platform with promising applications in biomedical monitoring and environmental analysis. In addition, some researchers have improved electrocatalytic performance by combining MOFs with 2D materials such as graphene or black phosphorus nanosheets to form novel composites. Cheng et al. [64] combined Mxene with MOFs to form a new 3D flower-like Cu-MOF/Mxene/GCE (glassy carbon electrode) material by taking advantage of Mxene’s high electrical conductivity and large specific surface area (Figure 5f). Owing to the large specific surface area of MXene, Cu-MOF was evenly dispersed on the surface, and the metal sites of MXene and Cu-MOF improved the catalytic ability of H_2_O_2_. The electrochemical tests showed that Cu-MOF/Mxene/GCE had a wide linear range of 1 µM to 6.12 mM at −0.35V with a detection limit of 0.35 µM. The material was also used to measure H_2_O_2_ in milk and serum with good recovery.

### 4.4. MOF Derivatives Based H_2_O_2_ Sensor

**Figure 6:**
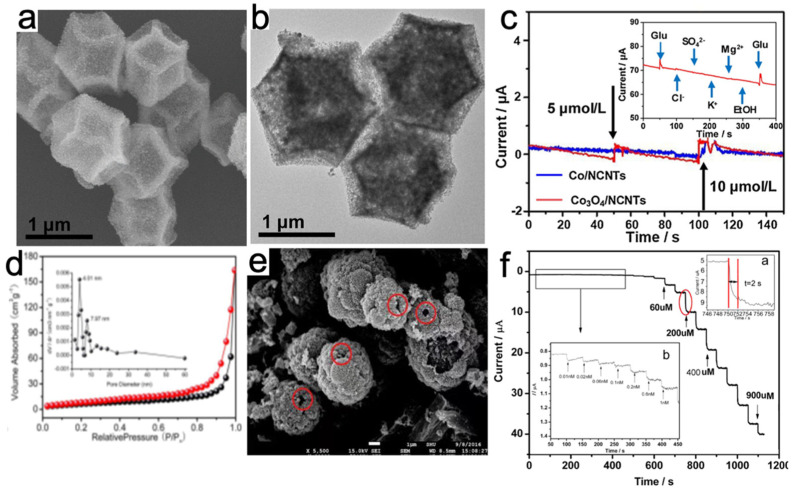
(**a**) Typical SEM images of Co_3_O_4_/NCNTs; (**b**) Typical TEM images of Co_3_O_4_/NCNTs; (**c**) Amperometric responses of Co/NCNTs and Co_3_O_4_/NCNTs with the addition of the same concentration H_2_O_2_ [144]; Copyright 2020, Elsevier. (**d**) Nitrogen adsorption-desorption isotherm of meso-CuCo_2_O_4_. Insets, respectively, show the crystal structure and distribution of pore size of meso-CuCo_2_O_4_; (**e**) SEM image of meso-CuCo_2_O_4_; (f) Current-time curve of different H_2_O_2_ concentrations on CuCo_2_O_4_/CPE in 0.2 M NaOH electrolyte at +0.5 V. Insets a and b respectively were response time of addition H_2_O_2_ and current-time curve for a low concentration H_2_O_2_ [145]. Copyright 2018, Elsevier.

MOFs are often treated as self-sacrificing metal-organic precursors by post-treatment or high-temperature pyrolysis to construct well-defined heteroatom-doped carbonaceous microstructures with specific surface properties. A variety of typical 3D MOFs such as Prussian Blue, MOF-5, ZIF-67, and HKUST (Hong Kong university of science and technology, also called MOF-199)-1 are widely used as precursors or templates to build novel energy storage structures [135,136,137,138,139,140]. Furthermore, it has been reported that the carbon hybridization of transition metal oxides can not only improve the electrical conductivity of the catalyst, but also effectively prevent the aggregation of the catalyst. Therefore, transition metal oxides are often used as precursors for MOF derivatives [141,142,143]. Firstly, transition metal oxides are often used to provide active sites for enzyme-free detection and improve the activity of catalysts. Secondly, in situ formed CNT framework can improve the electronic conductivity, which is beneficial to the mass transfer of target molecules. Thirdly, the encapsulated carbon shell can effectively immobilize the oxidized nanoparticles, thereby inhibiting their aggregation. Qin’s [144] group successfully pyrolyzed the MOFs and fabricated hollow frameworks of Co_3_O_4_/n-doped carbon nanotubes (Co_3_O_4_/NCNTs) in air. It indicated that Co_3_O_4_ nanoparticles supplied active sites for the enzyme-free detection of H_2_O_2_, and the in situ formed carbon nanotube framework enhanced the electronic conductivity and accelerated the mass transfer of target molecules (Figure 6a). The encapsulated carbon shell could potently immobilize the oxidized nanoparticles, thus inhibiting their aggregation (Figure 6b). The established hollow frameworks exhibited excellent bifunctional detection capability with high sensitivity and low detection limitation for H_2_O_2_ (87.40 μA (mmol/L)^−1^ cm^−2^, 1 mmol/L) and glucose (5 mmol/L) (Figure 6c). This provides an effective idea for the establishment of non-enzymatic sensors with multifunctional detection for biological applications. In addition to the Co_3_O_4_/NCNTs hollow frameworks, Cui et al. [145] successfully synthesized hollow mesoporous CuCo_2_O_4_ (meso-CuCo_2_O_4_) microspheres and utilized them in both H_2_O_2_ sensors and glucose biofuel cells (GFCs) for the first time (Figure 6d,e). Meanwhile, the nitrogen adsorption-desorption isotherm of meso-CuCo_2_O_4_ was a type IV isotherm, confirming the existence of mesoporous structure and the intrinsic high specific surface area. On this basis, the inherently high catalytic activity of mesophase Cuco_2_o_4_ exhibits high sensitivity and low detection limit (3 nM) for H_2_O_2_ (Figure 6f). This further demonstrates that tunable porous structure can be constructed through MOFs template sacrificial method. Meanwhile, more metals active sites can be loaded with the increased surface area. It should be noted is that the calcination temperature needs to be preciously controlled, in order to prevent the potential issues of agglomeration and resulting reduced catalytic activity.

## 5. Conclusions and Outlook

**Figure 7:**
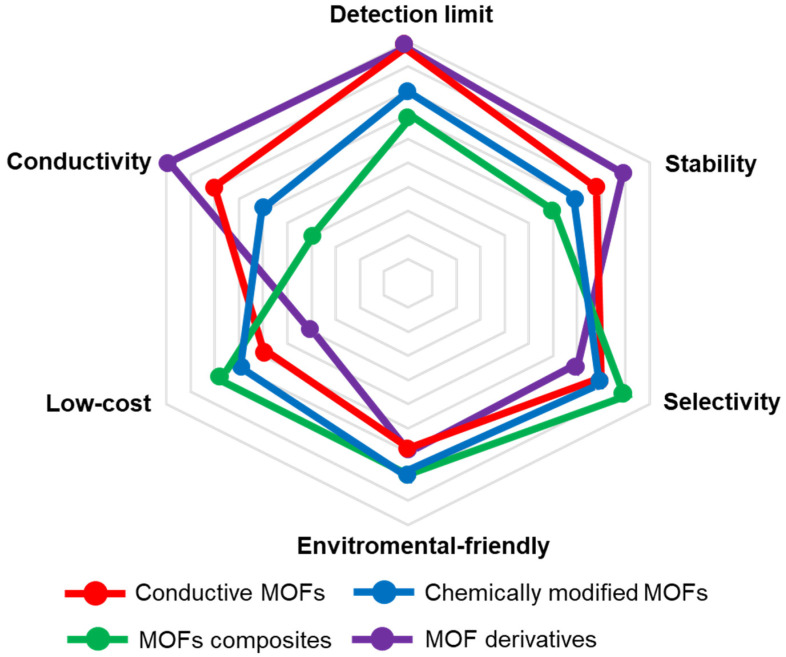
The pros and cons (such as detection limit, conductivity, stability, selectivity, low-cost and environmental-friendly) of the introduced catalysts for H_2_O_2_ electrochemical sensor (The outside is better, the inside is worse).

In summary, we listed the advantages and disadvantages (such as conductivity, catalytic activity, stability, selectivity cost and environmental friendliness) of conducting MOFs, chemically modified MOFs, MOFs composites and MOF derivatives (Figure 7). We consider that the ideal electrochemical sensors for H_2_O_2_ need to have the features of high conductivity, excellent catalytic activity, high selectivity, long-time stability, low cost, and environmental friendliness.

(i) Ultra-low detection limit

In order to reduce the detection limit of the sensor, it will be crucial to improve the electrocatalytic activity of the catalyst. Correspondingly, the following strategies can be adopted to improve it: a. Constructing a hollow porous structure not only helps to increase the specific surface area to expose more active sites, but also gives more 3D electron transfer channels; b. Establishing a conductive network improves the conductivity of the catalyst and facilitates electron transfer; c. Composite strategy of coalescing conductive materials or materials with high activity for the decomposition of H_2_O_2_ enhances conductivity and catalytic activity.

(ii) Long-term stability

It remains a huge challenge to achieve the long-term monitoring of H_2_O_2_ with sensors without any decrease in activity. In general, catalyzing over a long period of time can lead to the structural changes of materials and deactivation of catalytic sites. To solve this problem, catalysts can be grown directly on highly corrosion-resistant conductive substrates (such as GR nanosheets, conductive glass, Ni foam, etc.)

(iii) Large-scale production

Large-scale production of cheap and efficient H_2_O_2_ electrochemical sensors is of great significance for practical applications. However, the development process from basic research to commercialization is seriously hindered by the high price of some raw materials, synthesis processes and few synthesis methods. Therefore, new synthesis methods such as electrospinning, spray drying and Langmuir-Blodgett are needed to achieve precise control and mass production of electrocatalysts.

Overall, the development of highly sensitive and tolerant electrochemical sensors for H_2_O_2_ is an important and challenging topic in the field of detection. Even in the near future biological enzymes are also currently the best choice for detecting H_2_O_2_, but this is beyond the scope of this review. We hope that this review can provide some meaningful inspirations for the design and fabrication of MOFs based H_2_O_2_ electrochemical sensors. It is no doubt that the development of H_2_O_2_ electrochemical sensors with comprehensive performance and low cost will make significant contributions and breakthroughs in biological, medical and other fields.

## Figures and Tables

**Table 1:** Recently reports of H_2_O_2_ electrochemical sensors.

Electrochemical Sensor	Electrolyte	Detection Limit (μM)	Linear Range	Practical Application	Reference
**Conductive MOF** **s based** **H_2_O_2_ sensors**					
[Co_3_(HHTP)_2_]*_n_*	0.1 M NaOH	2.9	-	-	[2]
[Co_3_(HOB)_2_]*_n_*	0.1 M NaOH	0.00308	-	-	[36]
[Cu(adp)(BIB)(H_2_O)]*_n_*/GC	0.1 M KOH	0.068	0.1 μM–2.75 μM		[53]
2D Co-MOF	0.1 M KOH	0.69	0.5 μM–832.5 μM		[54]
FePc-CP NSs	0.1 M PBS	0.017	0.1–1000 μM	A549 live cells, Orange juice and beer	[55]
Co-MOF/TM	0.1 M PBS	0.25	1–13,000 μM	A549 cells	[56]
CuMOFs@FeP-pSC_4_-AuNPs	10 mM PBS	47	0.5–2.5 mM	Cancer cells	[57]
NENU5	0.1 M PBS	1.03	10–50,000 μM	-	[58]
CuCo-BDC/GO	0.1 M PBS	0.069	100 nM–3.5 mM	Human serum samples	[59]
HKUST-1/GCE	0.1 M PBS	0.68	2 μM–3 Mm and 3–25 mM	Milk sample	[60]
**MOF composites based H_2_O_2_ sensors**					
MIL-53-CrIIIAS/GCE	0.1 M NaOH	3.52	25–500 mM,	-	[54]
Ni(II)-MOF/CNTs nanocomposites	0.1 M NaOH	2.1	0.01–51.6 mM		[61]
MNPs@Y-1, 4-NDC-MOF/ERGO	0.1 M PBS	0.18	4–11,000 μM	A549 cells	[62]
Ni–MOF nanosheets/Hemin	0.1 M PBS	0.2	1–400	Human serum samples	[9]
GCE/GO/poly(CoTBIPc)	0.1 M PBS	0.6	2–200 μM	-	[3]
A-Ni_1_Mo_0.5_-MOFs@AAC	PSB	0.185	-	-	[63]
CuCo-BDC/GO	0.1 M PBS	0.069	100 nM -3.5 mM	Diluted human serums	[59]
CuMOF/MXene/GCE	0.1 M PBS	0.35	1 µM–6.12 mM	Serum	[64]
Cu-TCPP MOF/Cu_5.4_O	0.1 M PBS	0.13	0.0001- 0.59 mM and 1.59–20.59 mM	Living cells	[65]
Cu-MOF@S-Gr	0.1 M PBS	0.0113 ± 0.00004	0.1–3 μM	Tap water	[66]
Au–Pd@UiO-66-on-ZIF-L/CC	0.01 M PBS	0.0212	1 μM–19.6 mM	A549 cells	[67]
Cu@BDC(NH2)@2-MI	0.1 M PBS	0.97	10 μM–13.28 mM		[68]
MnO_x_	0.2 M PBS	0.000232	0.000696–742 μM	Human serum and milk sample	[69]
NCNT MOF CoCu	0.1 M PBS	0.206	0.05–3.5 mM	Serum samples	[70]
Ag-Bi BDC (s) MOF/GCE	0.1 M PBS	0.020.1	10 μM–5 mM and 5 mM–145 mM	THP-1 and AtT-20 cancer cells	[71]
**MOF derivatives based H_2_O_2_ sensors**					
AuPt/ZIF-8−rGO	0.1 M PBS	0.019	0.1–18,000 μM	Human serum	[72]
MOF-Au@Pt nanoflowers	PBS	0.086	0.8 μM–3 mM	Suspension of living cell	[32]
Co-NC RDCs	0.1 M PBS	0.143	0.001–30 mM		[73]
MIL-101(Fe)@Fe_3_O_4_/NGCE	0.1 M PBS	0.15	0.001–0.01 mM	Human blood plasma	[74]
Co (4%)–N/CNS	0.4 M PB	0.00618	1–500 μM and 500 μM–0.1 M	Human serum	[75]
Co-NPs/NCs	0.01 M PBS	0.12	10–2080 μM and 2080–11,800 μM	Human serum sample	[76]
Cu-MoO_2_-C	PBS	0.16	0.25–6.25 mM		[77]
ZnO@ZIF-8	0.1 M PBS	3	20–11,550 μM		[78]
Co_3_O_4_@CNBs	0.01 M PBS	0.00232	10 nM–359 μM	HUVEC cells and 4T1, A549 cancer cells	[79]

## Data Availability

Not applicable.
